# A Turn-On Fluorescent Chemosensor for Cyanide Ion Detection in Real Water Samples

**DOI:** 10.3389/fchem.2022.923149

**Published:** 2022-07-18

**Authors:** Qing Shi, Shou-Ting Wu, Lingyi Shen, Tao Zhou, Hong Xu, Zhi-Yong Wang, Xian-Jiong Yang, Ya-Li Huang, Qi-Long Zhang

**Affiliations:** ^1^ School of Public Health, The Key Laboratory of Environmental Pollution Monitoring and Disease Control, Ministry of Education, Guizhou Medical University, Guiyang, China; ^2^ The Second Affiliated Hospital of Guizhou University of Traditional Chinese Medicine, Guiyang, China

**Keywords:** synthesis, crystal structure, fluorescent probe, cyanide ion, real sample detection

## Abstract

We have designed and synthesized a novel simple colorimetric fluorescent probe with aggregation-induced emission (AIE) properties. Probe 5-(4-(diphenylamine)phenyl) thiophen-2-formaldehyde **W** exhibited a turn-on fluorescent response to cyanide ion (CN^−^), which induces distinct visual color changes. Probe **W** exhibited a highly selective and sensitive ratiometric fluorescence response for the detection of CN^−^ over a wide pH range (4–11) and in the presence of common interferents. The linear detection of CN^−^ over the concentration range of 4.00–38.00 µM (*R* ^2^ = 0.9916, RSD = 0.02) was monitored by UV-Vis absorption spectrometry (UV-Vis) with the limit of detection determined to be 0.48 µM. The linear detection of CN^−^ over the concentration range of 8.00–38.00 µM was examined by fluorescence spectrophotometry (*R* ^2^ = 0.99086, RSD = 0.031), and the detection limit was found to be 68.00 nM. The sensing mechanisms were confirmed by ^1^H NMR spectroscopic titrations, X-ray crystallographic analysis, and HRMS. Importantly, probe **W** was found to show rapid response, high selectivity, and sensitivity for cyanide anions in real water samples, over the range of 100.17∼100.86% in artificial lake water and 100.54∼101.64% in running water by UV-Vis absorption spectrometry, and over the range of 99.42∼100.71% in artificial lake water and 100.59∼101.17% in running water by fluorescence spectrophotometry. Importantly, this work provides a simple and effective approach which uses an economically cheap and uncomplicated synthetic route for the selective, sensitive, and quantitative detection of CN^−^ ions in systems relevant to the environment and health.

## Introduction

Cyanide is a highly toxic substance and an important raw material. It is widely used in industrial production in areas such as metallurgy. It is also widely used in extraction processes associated with synthetic fibers, resins, herbicides, and gold. Among the numerous anions, CN- has attracted wide attention because of its uses and rapid toxicity (Chen et al., 2015), and because of this, unavoidable cyanide emissions are a potential threat to the environment and human beings. Even trace amounts of CN- are easily absorbed by the human lungs, gastrointestinal tract, or skin, causing neurological disorders and respiratory diseases that eventually lead to death (Li et al., 2016). According to the World Health Organization (WHO) and GB 5749-2006, the maximum allowable value of CN- in drinking water is 1.00 mg/L (about 1.90 μM) (Sanitary standards for drinking water; Chemical contaminants in food around the world, 2009). For humans, the lethal dose of cyanide is 0.50 mg/kg to 3.50 mg/kg (body weight) (Spencer and Sigler, 1983; Shan and Mousty, 2004; Ishii et al., 1998). The sources of cyanide in food are complex. In food plants, cyanide is usually combined with sugar molecules to form cyanosides. For example, tapioca contains cyanoglucoside, while amygdalin contained in almonds is also a cyanoglucoside. There are also some plants that present cyanide in the free form, such as edible fungi, mushrooms, and fungi, which have been detected to have high levels of cyanide. The content of cyanoside ligand in plants may be related to the plant's genetic gene, environment, location, climate, and soil (Song et al., 2020). Cyanide is easily absorbed by the body and can enter the body through the mouth, respiratory tract, or skin. Cyanide entering the stomach can be immediately hydrolyzed into HCN and absorbed under the disintegration of gastric acid. After HCN enters the blood circulation, the iron-containing pro-group of cytochrome oxidase in the blood will combine with CN^−^ to generate cytochrome oxidase of cyanide, resulting in the interruption of the respiratory chain and loss of the ability to transfer electrons, resulting in cell asphyxia and death. When the body ingests too much CN^−^, it can cause vomiting, convulsions, loss of consciousness, and in some cases, death (Xu et al., 2010; Liu and Shao, 2011; Kim and Park, 2012). Given that the solubility of cyanide in lipids is relatively large, it is the central nervous system that is first harmed, and moreover, because of the increased sensitivity of the respiratory center, respiratory failure is the main cause of death from acute cyanide poisoning (Lin et al., 2013). Therefore, the toxic effects of CN^−^ on the environment and the biological harm it can do cannot be ignored. With this in mind, the detection and monitoring of CN^−^ in environmental and life sciences has become an important application .

At present, the detection mechanisms for CN^−^ using reported photochemical sensors mainly include a nucleophilic addition reaction (Wang et al., 2016; Beneto and Siva, 2017), hydrogen bonding (Kumar et al., 2019; Zhou et al., 2016), substitution of metal complexes (Park et al., 2014; Wang et al., 2015; Qu et al., 2016; Zhou et al., 2019; Arivazhagan et al., 2016), and deprotonation (Chen et al., 2015; Zhu et al., 2015; Ebaston et al., 2016; Li et al., 2016; Zhang et al., 2016). As a kind of photochemical sensor, the sensitivity of a fluorescence sensor can reach the order of magnitude of 10-12-10-9, so it can reflect the change of chemical information by using the change of signal. Material molecules can absorb visible or ultraviolet light or cause electron transitions on energy levels, at which time the molecules will be excited to a relatively high electronic energy state. When the excited state transitions to the ground state, the system releases energy in the form of radiative transitions and generates fluorescence. The fluorescent chemical sensor has many advantages, such as its small size, low cost, is not affected by an external electromagnetic field, has high sensitivity, can be remote light, easy to detect, at the same time, it is fully automated, and does not need preprocessing. It can communicate between molecules and humans, and it can visually distinguish subparticles in subnanometer space and submillisecond time, and this has been favored by an increasing number of researchers in recent years (Ma and Dagupta, 2010; Lou et al., 2012; Afshani et al., 2016; Chao et al., 2016; Sun, 2016).

In this study, we have designed and developed fluorescent probe **W** which possesses strong specificity and high sensitivity for the detection of cyanide. It can detect CN^−^ in a water environment by UV-Vis absorption spectroscopy as well as by fluorescence spectroscopy.

## Experimental

### Equipment and Reagents

Inova-400 MHz NMR spectrometer (Varian Company, United States), VGT-2227QTD type ultrasonic instrument (Shenzhen Gute Hongye Machinery Equipment Co., Ltd., China), CP214 electronic balance (Shanghai Aohaus Instrument Co., Ltd., China), Cary Eclipse type fluorescence spectrophotometer (Varian Company, United States), UV-visible spectrophotometer of UV-2600 (Suzhou Dao Jin Instrument Co., Ltd., China), pH meter of pHS-25 (Chengdu Century Ark Technology Co., Ltd., China), and Bruker Smart Apex single crystal diffractometer (Bruker AXS Company, Germany) were obtained.

5-(4-(diphenylamine)phenyl)thiophen-2-formaldehyde, 2,3,3-trimethylindole, iodoethane, acetonitrile, anhydrous ethanol (EtOH), *n*-hexane, cyanide (NaCN), hydrochloric acid (HCl), anions, metal ions, and amino-containing small molecules such as glutathione (GSH) are commercially available and were purchased from Aladdin Reagent Co., Ltd. (Shanghai, China). All chemicals were of analytical grade and were used without further purification. All the water used for the preparation of the solutions in the experiment was ultrapure water (conductivity 18.2 MΩ cm, Youpu Super Pure Technology Co., Ltd. Sichuan, China).

### Synthesis of Compound 1a

The synthesis is briefly described as follows: 3.20 g (20 mmol) 2,3,3-trimethylindole and 3.10 g (20 mmol) iodoethane were mixed in 80 ml acetonitrile and stirred at 90 °C for 12 h. The solution was cooled to room temperature to give 1-ethyl-2,3,3-trimethylindole iodide (**1a**) as a red solid (5.23 g) in a yield of 83%. The molecular formula of compound **1a** is C_13_H_18_IN.

### Synthesis of Fluorescent Probe W

First, 0.36 g (1 mmol) 5-(4-(diphenylamine)phenyl)thiophen-2-formaldehyde and 0.63 g (2 mmol) compound **1a** were mixed in 40 ml acetonitrile and stirred at 90°C for 8 h. The solution was concentrated under pressure and recrystallized to obtain blue black powder (0.74 g) with a yield of 75%. The molecular formula of fluorescent probe **W** is C_36_H_33_IN_2_S. ^1^H NMR (400 MHz, CDCl_3_) *d* 8.51 (1H, d, *J* = 15.2), 7.63–7.40 (1H, m), 7.32 (1H, dt, *J* = 15.6, 7.8), 7.26 (1H, s), 7.15 (1H, ddd, *J* = 19.3, 11.7, 4.2), 7.05 (1H, d, *J* = 8.8), 4.79 (1H, m), 1.87 (1H, s), and 1.63 (1H, dd, *J* = 19.9, 12.6) (Supplementary Figure S1). ^13^C NMR (101 MHz, DMSO-d_6_) *d* 179.77, 154.54, 148.96, 146.20, 146.04, 143.51, 140.52, 140.33, 138.23, 129.85, 129.05, 128.73, 127.47, 125.40, 125.26, 124.42, 123.03, 121.28, 114.48, 108.79, 51.71, 40.15, 25.77, and 13.53 (Supplementary Figure S2). HRMS calculated: 525.2359; found: 526.2408 (Supplementary Figure S3).

### X-Ray Crystallography

Crystallographic data for ligand **W** were collected on a Bruker APEX 2 CCD diffractometer with graphite-monochromated Mo Kα radiation (*λ* = 0.71073 Å) in the ω scan mode (Krause et al., 2015). The structure was solved by a charge flipping algorithm and refined by full-matrix least-squares methods on F2 (Sheldrick, 2015). All esds were estimated using the full covariance matrix. Further details are presented in Supplementary Table S1.

### General Methods for Optical Tests

In total, 10.52 mg (20 μM) of probe **W** was dissolved in 10.00 ml of EtOH solution to prepare a 2.00-mM reserve solution. Then the nitrates of the metal ions, the sodium salt of anions, and small amino molecules (Ag^+^, Al^3+^, Cd^2+^, Co^2+^, Cr^3+^, Cu^2+^, Fe^3+^, Hg^2+^, K^+^, Li^+^, Mg^2+^, Na^+^, Ni^2+^, Pb^2+^, Zn^2+^, AcO^−^, Br^−^, C_2_O_4_^2−^, ClO_4_^−^, Cl^−^, CN^−^, CO_3_^2−^, F^−^, H_2_PO_4_^−^, HCO_3_^−^, HSO_3_^−^, HPO_4_^2−^, I^−^, NO^2−^, NO_3_^−^, SCN^−^, PO_4_^3−^, S_2_O_3_^2−^, SO_3_^2−^, SO_4_^2−^, GSH, Hcy, H_2_NCONH_2_, and Cys) were accurately weighed and dissolved in 10.00 ml of the PBS to form 10 mM ion stock solutions. The NaCN solution was accurately extracted with a 0.10 ml pipette gun and diluted with 10.00 ml of PBS solution to form 10 mM ion reserve solutions. The preparation method of the PBS solution (10 mM) was as follows: 23 g of PBS powder was weighed and dissolved in 2 L ultrapure water at pH 7.20–7.40.

## Results and Discussion

### Synthesis

**SCHEME 1:**
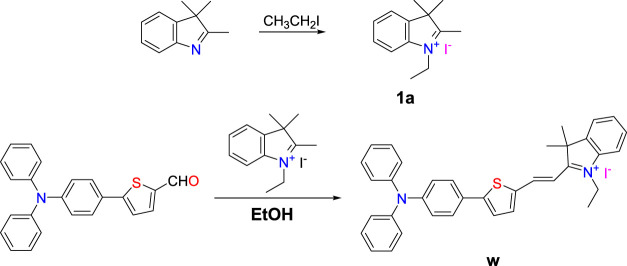
Synthetic route to probe **W**.

New and highly sensitive fluorescent probe **W** was obtained from 5-(4-(diphenylamine) phenyl)thiophen-2-formaldehyde and 1-ethyl-2,3,3-trimethylindole iodide (compound **1a**, synthesized from 2,3,3-trimethylindole and iodoethane) by a simple two-step reaction (Scheme 1). The molecular structure was characterized by ^1^H NMR spectroscopy, HRMS, and single crystal X-ray diffraction. Probe **W** exhibited excellent solubility in common organic solvents (such as methanol, ethanol, and EtOH) and possessed good acid and alkali resistance over the pH range of 4–11 over 24 h (Supplementary Tables S2, S3). The thickness of the plate is 1 cm, that is, the thickness of the liquid layer. The concentration of CN^−^ was 40.00 μM, εmax = 15197.5 L mol^−1^·cm^−1^, and *λ*(abs) = 382 nm.

### Determination of Optimum Experimental Conditions

Anion fluorescent probes are mainly used in the fields of biology, medicine, and food monitoring. If they can be utilized in aqueous solution, they will have far wider application value. In addition, a buffer solution can be used to control the pH value of an aqueous solution, making the results of identification more reliable (Chan et al., 2012; Lee et al., 2015b). Therefore, in our experimental exploration, we have determined the optimum water content for probe use.

**FIGURE 1:**
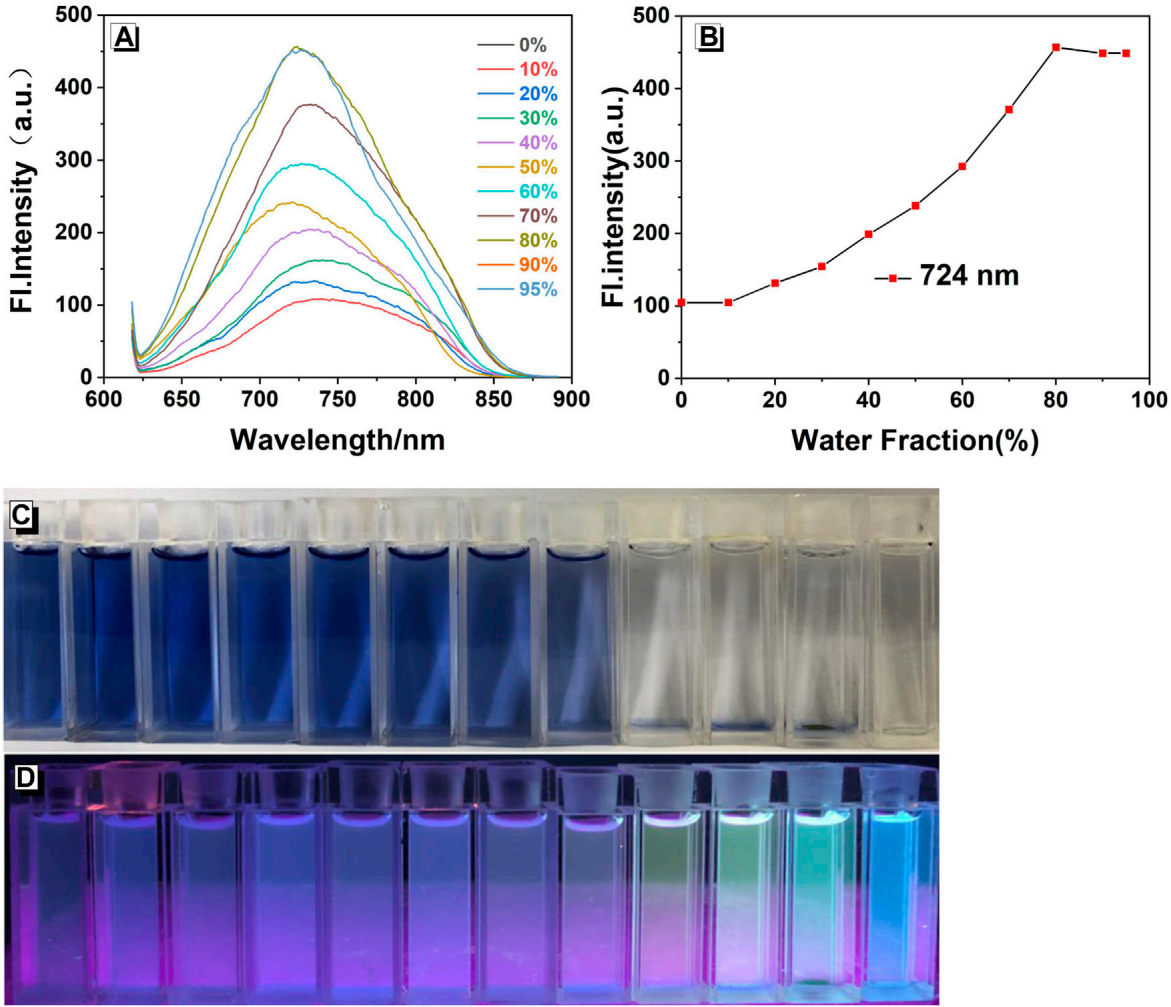
**(A)** Fluorescence spectra of W (20 μM) in EtOH/water mixtures with different water fractions (λex/λem = 378 nm/724 nm, slit: 5/5 nm, and voltage: 800 V). **(B)** Plots of fluorescence intensity at 724 nm. Photographs in EtOH/water mixtures with different water fractions taken under **(C)** natural light and **(D)** 365 nm UV irradiation.

As shown in Figure 1, compound **W** emits a near-infrared emission in the ethanol solution, with *λ*_max_ _em_ = 724 nm. As the water content (FW) increased from 0 to 30%, the fluorescence intensity hardly changed. Subsequently, when the water fraction (*f*w) reached 70%, some particles could be observed in the mixture, which showed a bright blue-green light under 365 nm UV irradiation. The fluorescence spectrum also exhibits a similar phenomenon: the fluorescence intensity at 80% is much higher than that at *f*w = 0–30%. When the water fraction reached 80%, the fluorescence intensity of the solution attained the maximum value (457 a.u.) with an approximate 3.37-fold increase *versus* that of the pure solution. We used the relative quantum yield measurement method, using the formula: *F=KI*_*0*_ *AΦ*. The quantum yield of the ethanol solution reached Φ *f* = 18.4%. Thus, probe **W** is a chromophore with aggregation-induced enhanced emission (AIEE) properties. Since fluorescent probe **W** is insoluble in water and insensitive to the recognition and detection of ions or molecules at high water content, we chose the mixture EtOH/water (V_EtOH_/V_H2O_ = 3:2) as the recognition environment.

**FIGURE 2:**
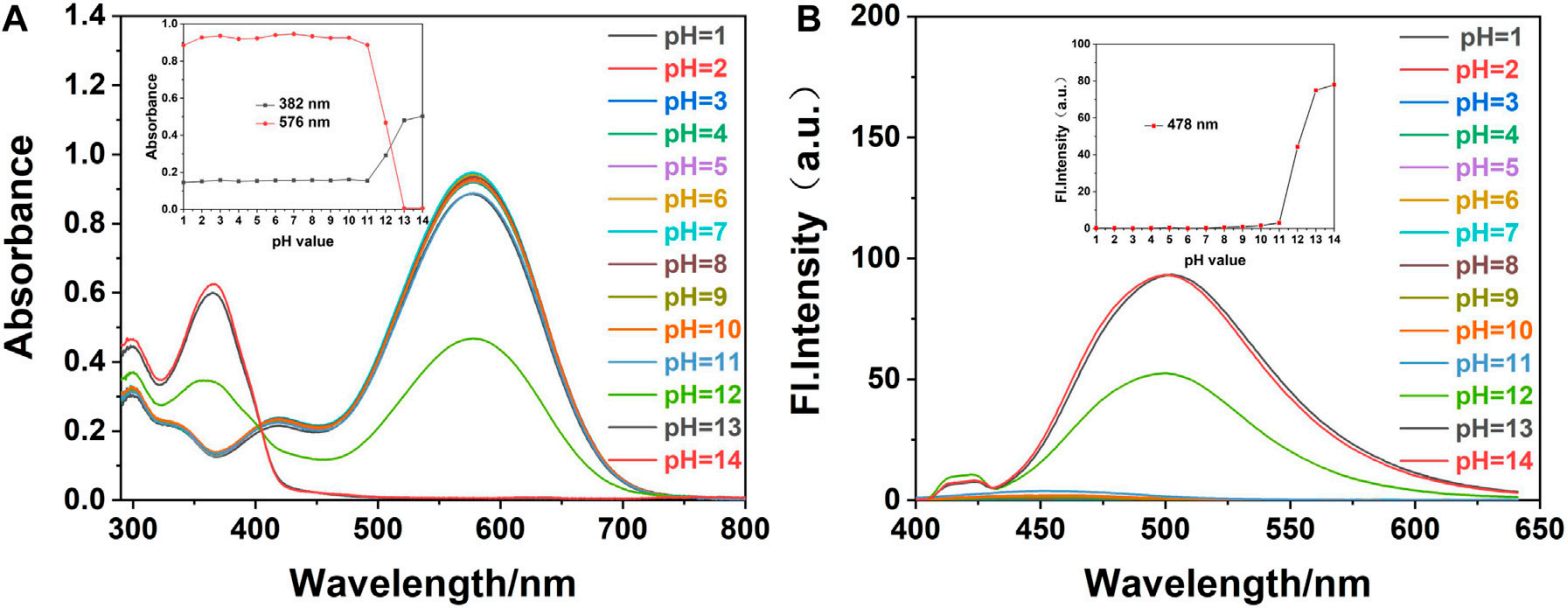
**(A)** The UV-Vis and **(B)** fluorescence spectra of fluorescence probe W (20 μM) in EtOH/water (VEtOH/VH2O = 3/2) at different pH values (λex/λem = 378 nm/479 nm, slit: 5/5 nm, and voltage: 500 v). Inset: **(A)** effect of different pH values on the absorbance of probe W at 382 and 576 nm. **(B)** Influence of pH values on fluorescence probe W at 478 nm.

**FIGURE 3:**
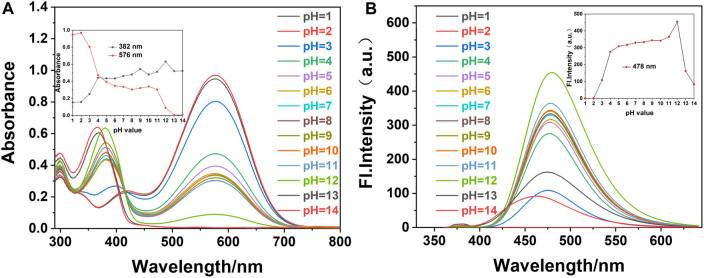
**(A)** UV-Vis and **(B)** fluorescence spectra of fluorescence probe W (20 μM) in EtOH/water (VEtOH/VH2O = 3/2) with the addition of CN^−^ (40 μM) at different pH values (λex/λem = 378 nm/478 nm, slit: 5/5 nm, and voltage: 500 v). Inset: **(A)** effect of different pH values on the absorbance of probe W with the addition of CN^−^ at 382 and 576 nm. **(B)** Influence of pH values on fluorescence probe W with the addition of CN^−^ at 478 nm.

The pH value of the environment is a critical parameter that may affect the selective sensitivity and detection limit of the probe (Feng et al., 2018). The UV-Vis absorption and fluorescence spectra of probe **W** (Figure 2) and cyanide (CN^−^) (Figure 3) identified by probe **W** were experimentally studied over the pH range of 1–14.

We added 1.20 ml of PBS solution of different pH values into a 3.00 ml colorimetric dish, and then added 0.03 ml of the probe reserve solution. The solution was made to constant volume with anhydrous ethanol to 3.00 ml, shaken well, and left to react completely. The effects of the different pH values on the probe were measured by a UV-Vis spectrophotometer and a fluorescence photometer. As shown in Figure 2, in the detection system composed of EtOH/water (V_EtOH_/V_H2O_ = 3/2, 10 mM PBS), when the pH was in the range of 1∼11, the maximum absorption peak of probe **W** in the UV-Vis spectrum was 576 nm, and the fluorescence intensity was very weak. When the pH was between 12 and 14, the absorption peak appeared at 382 nm, and the maximum emission in the fluorescence spectrum was at 478 nm. In this range, the absorbance and fluorescence intensity of probe **W** were only slightly affected by the pH.

We added 1.20 ml of PBS solution of different pH values into a 3.00-ml colorimetric dish, followed by 12.00 µL CN^−^ reserve solution and 0.03 ml of the probe reserve solution, and made the volume up to 3.00 ml with anhydrous ethanol. The system was shaken well and left to stand until the solution had fully reacted. The effect of pH on the interaction between probe **W** and CN^−^ was determined by a UV-Vis spectrophotometer and a fluorescence photometer. As shown in Figure 3, when CN^−^ (40 µM) was added to the probe solution and the pH value was 1-3, the maximum absorption peak of probe **W** in the UV-Vis spectrum was at 576 nm, and the fluorescence intensity was very weak in the detection system composed of EtOH/water (V_EtOH_/V_H2O_ = 3/2, 10 mM PBS). On increasing the pH value, the maximum absorption peak of probe **W** in the UV-Vis spectrum is at 382 nm and the maximum emission peak in the fluorescence spectrum is at 478 nm over the pH range of 4–11. The absorbance and fluorescence intensity decrease gradually when the pH was between 12 and 14.

In the experiment, we investigated the influence of water content and the pH value on probe **W** and the recognition and detection ability of **W**-CN^−^, and selected EtOH/water (V_EtOH_/V_H2O_ = 3/2, 10 mM PBS, and pH = 7.40) as the detection system conditions. We also tested the optical stability of probe **W** and **W**-CN^-^ mixture over time, and the results showed that **W** and **W**–CN^-^ complex reacted rapidly and remained stable over time (Supplementary Figures S4, S5).

### Anion Sensing Study

The high selectivity and sensitivity of the probe are key parameters for domestic water detection and *in vivo* research. Therefore, to test the detecting ability toward anions, probe **W** (20 μM) was exposed to many anions (such as AcO^−^, Br^−^, C_2_O_4_^2−^, ClO_4_^−^, Cl^−^, CN^−^, CO_3_^2−^, F^−^, H_2_PO_4_^−^, HCO_3_^−^, HSO_3_^−^, HPO_4_^2-^, I^−^, N_2_H_4_·H_2_O, NO^2−^, NO_3_^−^, SCN^−^, PO_4_^3−^, S_2_O_3_^2-^, SO_3_^2-^, SO_4_^2−^, and [A]^n−^ = 80 μM), metal ions (such as Ag^+^, Al^3+^, Cd^2+^, Co^2+^, Cr^3+^, Cu^2+^, Fe^3+^, Hg^2+^, K^+^, Li^+^, Mg^2+^, Na^+^, Ni^2+^, Pb^2+^, Zn^2+^, and [M]^n+^ = 80 μM), and amino-containing small molecules (such as GSH, Hcy, H_2_NCONH_2_, Cys, and [M]^n+^ = 80 μM) in mixtures of EtOH and water (V_EtOH_/V_H2O_ = 3/2 and pH = 7.40).

**FIGURE 4:**
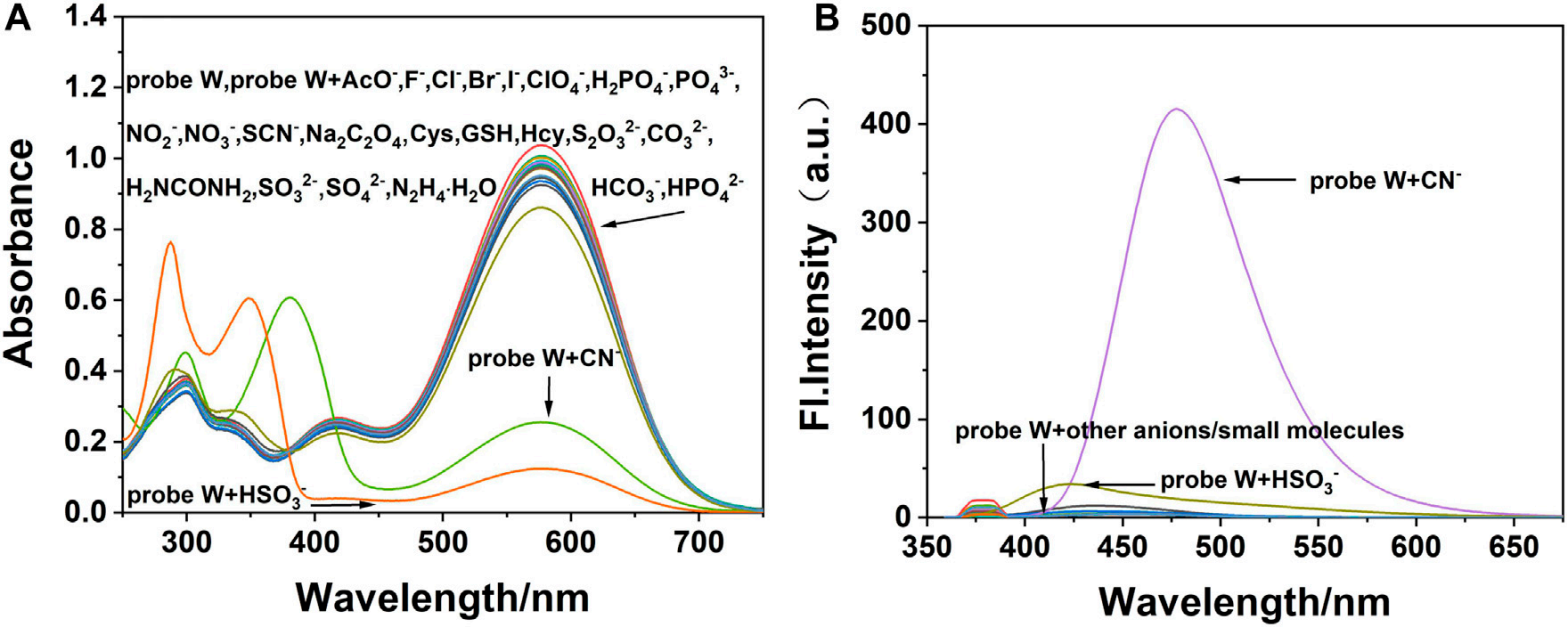
**(A)** UV-Vis and **(B)** the fluorescence spectra (λ ex/λ em = 378/478 nm) of fluorescence probe W interacting with different anions and amino-containing small molecules (slit: 5/5 nm, voltage: 500 V).

As shown in Figure 4, HSO_3_^−^ and CN^−^ cause the color of the solution to change *via* naked-eye observation (Supplementary Figure S6) after adding the anions and amino-containing small molecules into the solution of probe **W**. When CN^−^ was added, the maximum absorption peak at 576 nm was significantly reduced and a new peak appeared at 382 nm in the UV-Vis absorption spectrum. The maximum emission peak appeared at *λ*_em_ = 478 nm in the fluorescence spectrum, and the fluorescence intensity was significantly enhanced. When HSO_3_^−^ was added, the maximum absorption peak at 576 nm in the UV-Vis spectrum decreased significantly, and new absorption peaks appeared at 288 and 350 nm. The fluorescence intensity at 421 nm was slightly enhanced. Therefore, HSO_3_^−^ and CN^−^ can be selectively identified and detected by the fluorescence emission intensity. The absorption spectra and fluorescence spectra of the **W**–anion mixture indicated that probe **W** exhibits good selectivity toward CN^−^, while other cations or anions (Supplementary Figures S6A,B) had little impact on the optical behavior of probe **W**. On the other hand, under a 365-nm UV lamp, only the **W**–CN^-^ mixture led to the emission light enhancing dramatically (Supplementary Figures S6, S7D).

**FIGURE 5:**
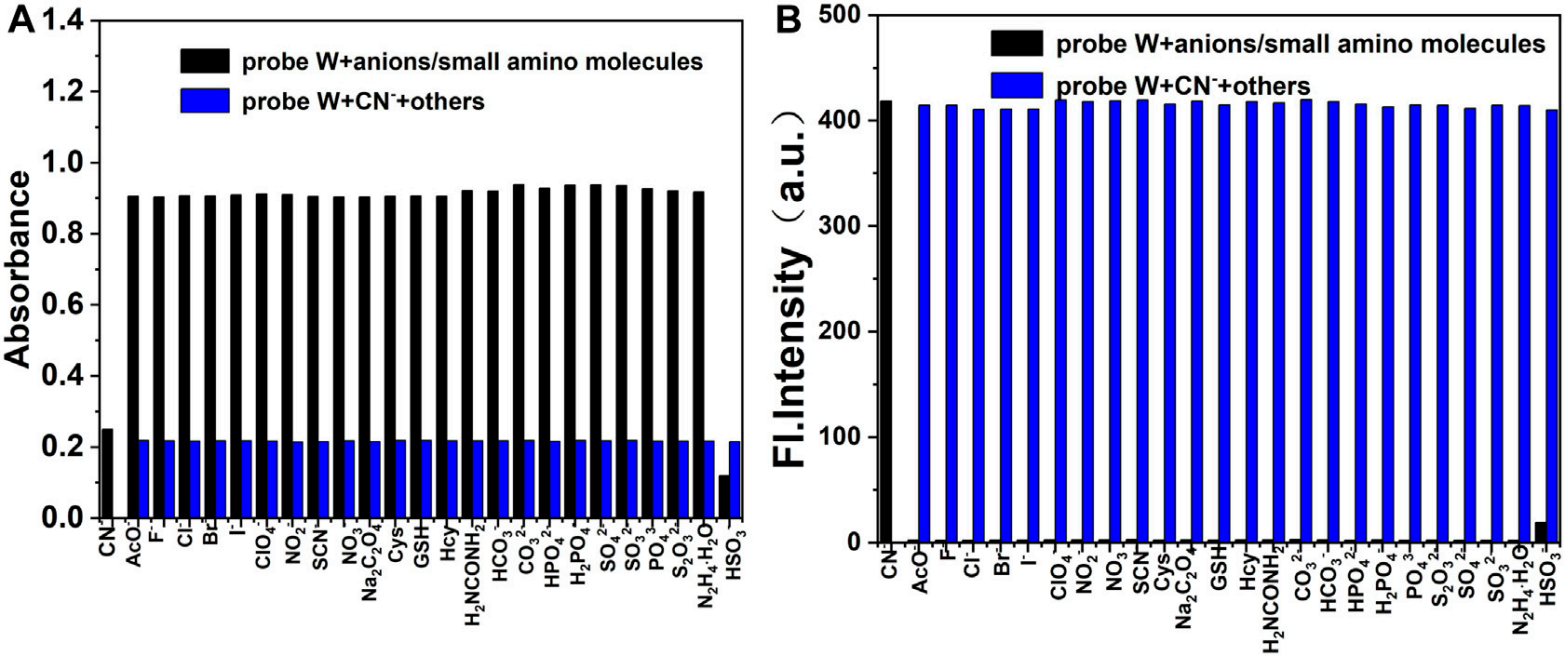
Bar diagram of the competitive experiments of various anions and amino-containing small molecules on the absorbance **(A)** and fluorescence intensity **(B)** of the probe/CN^−^ complex in buffer solution (λex/λem = 378 nm/478 nm, slit: 5/5 nm, and voltage: 500 V).

**FIGURE 6:**
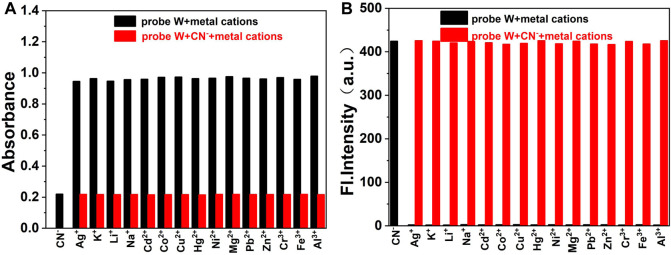
Bar diagram of the competitive experiments of various metal cations on the absorbance **(A)** and fluorescence intensity **(B)** of the probe/CN^−^ complex in buffer solution (λex/λem = 378 nm/478 nm, slit: 5/5 nm, and voltage: 500 V).

Furthermore, in order to investigate the competitive detection ability of probe **W** in the presence of other interferences, an ionic interference test was carried out. By observing the changes of absorbance at the absorption peak of 576 nm and fluorescence intensity at *λ*_em_ = 478 nm, the mixed solution containing CN^−^ was compared with the solution without CN^−^. We found that the maximum absorption peak in the UV spectrum decreased slightly after the addition of HSO_3_^−^, but the absorbance and fluorescence intensity did not change significantly after the addition of other anions, cations, and amino-containing small molecules (Figures 5, 6). The results showed that the coexisting cations/anions/amino-containing small molecules had limited influence on the detection of CN^−^. Thus, the interference experiments indicated that the probe displays a high specificity, strong anti-interference ability, and selectivity for detecting CN^−^ ions.

### Titration and Detection Limits

**FIGURE 7:**
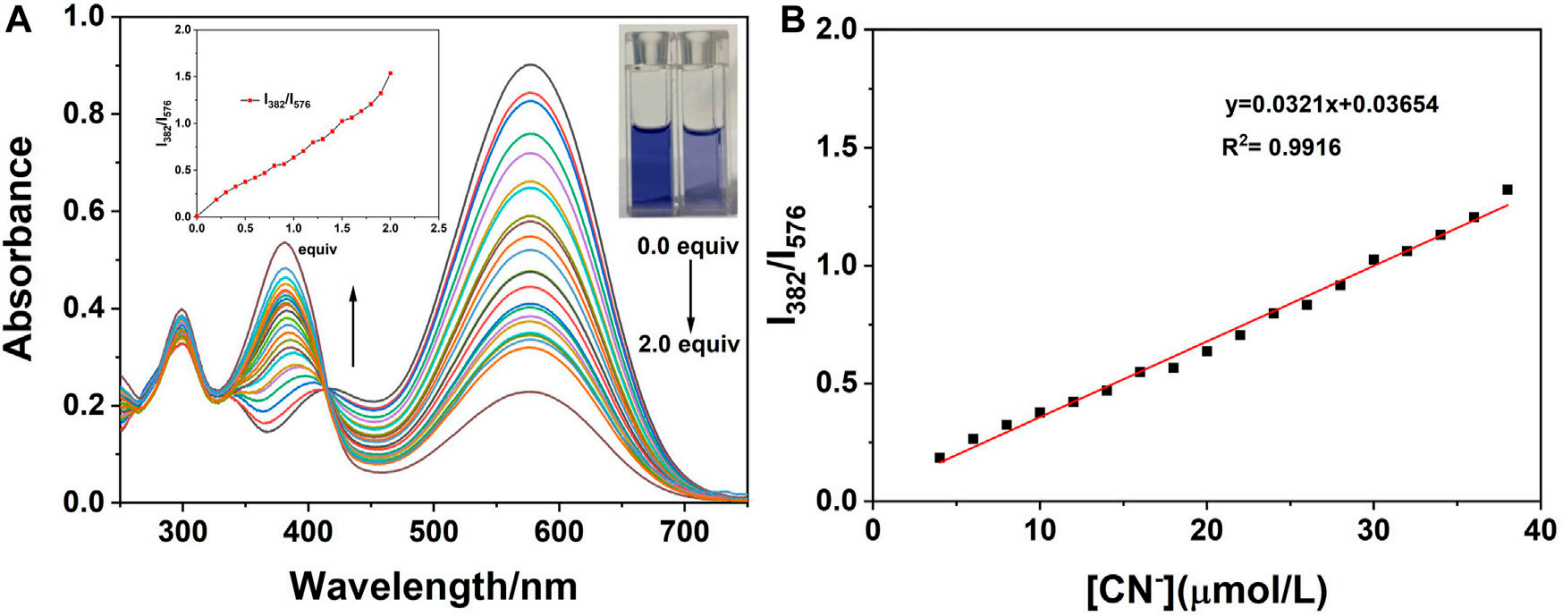
**(A)** UV-Vis absorption spectra on the addition of CN^−^ to the probe; **(B)** linear relationship between the ratio of absorbance at 382–576 nm and the concentrations of CN^−^ (4∼38 μM). Inset: the change curve of the ratio of absorbance at 382–576 nm with different concentrations of CN^−^; photograph of the solutions under illumination with sunlight showing the change of the solution after the titration is complete.

On the basis of the aforementioned experimental conditions, the UV titration experiments were performed with progressive addition of CN^−^ and the results are presented in Figure 7. As can be seen from the figure, on the addition of CN^−^ ions, the absorption peak at 576 nm gradually decreased and that at 382 nm gradually increased. When the concentration of CN^−^ reaches twice that of the probe concentration, it tends to be stable. In addition, when the concentration of probe **W** varied from 4 to 38 μM, it showed a good linear relationship with CN^−^ ions (y = 0.0321x + 0.03654, *R* ^2^ = 0.9916, RSD = 0.02). Herein, the detection limit was calculated by using the data of the UV titration experiments. According to the IUPAC method, namely, under the same conditions, 10 groups of blank samples were tested without cyanide, and then the standard deviation (SD) was calculated according to the ratio of the absorption peak at 382 nm to the absorption peak at 576 nm. Then, according to the formula, the detection limit = 3SD/S, where S is the slope of the linear relationship during the UV titration, and the detection limit of probe **W** for CN^−^ was calculated to be 0.48 μM. Compared with other CN^−^ probes (Supplementary Table S4), probe **W** had the advantage of a lower detection limit.

**FIGURE 8:**
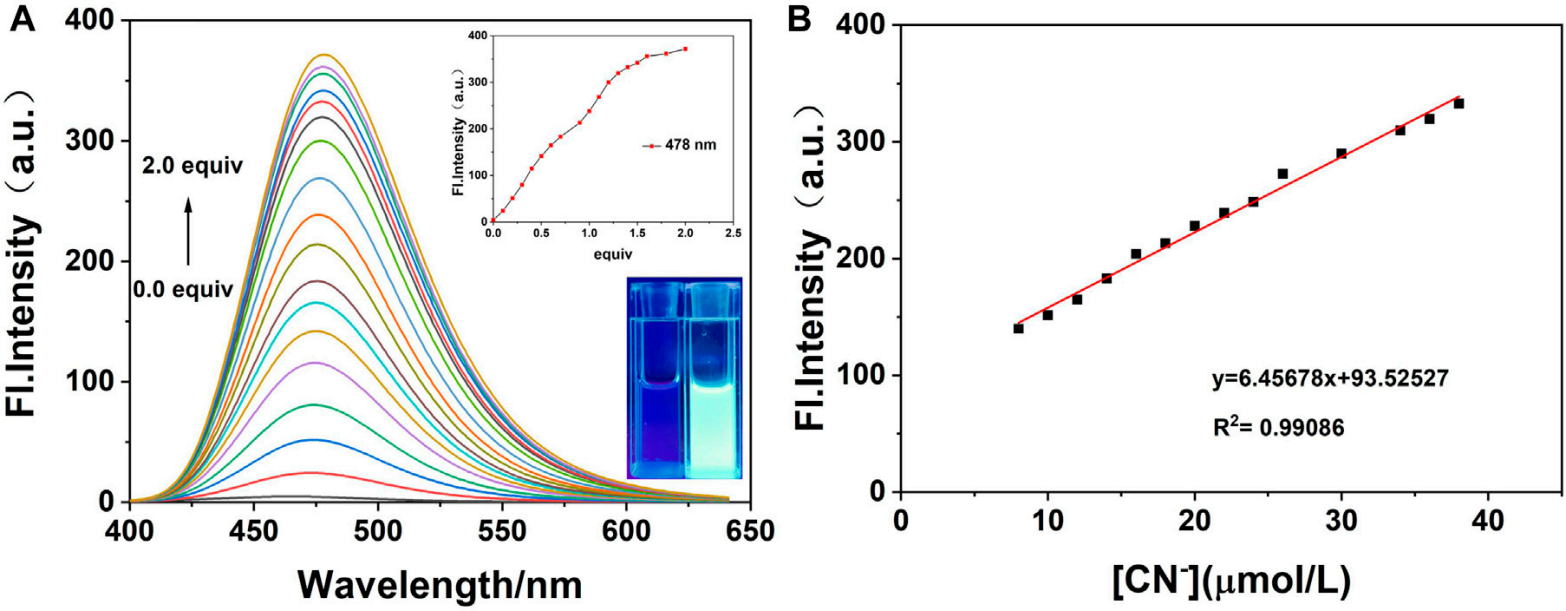
**(A)** Fluorescence spectra on the addition of CN^−^ to probe W (20 μM); **(B)** linear curve of fluorescence intensity of probe solution at λmax em = 478 nm and concentration of CN^−^ (8∼38 μM). Inset: curve of fluorescence intensity at λmax em = 478 nm with different concentrations of CN^−^; photograph of the fluorescence change under the irradiation of 365 nm UV lamp after the titration is complete.

On the basis of the aforementioned experimental conditions, the fluorescence titration experiments were performed with progressive addition of CN^−^ and are presented in Figure 8. The figure shows that the fluorescence intensity of probe **W** increased gradually at *λ*_max em_ = 478 nm on the addition of CN^−^ ions. In addition, when the concentration of probe **W** varied from 8 to 38 μM, it showed a good linear relationship with CN^−^ ions (y = 6.45678x + 93.52527, *R* ^2^ = 0.99086, and RSD = 0.31). Using the experimental data of the fluorescence titration, the detection limit was calculated according to the IUPAC method, that is, 10 groups of blank samples were detected in the absence of cyanide under the same conditions, and then the standard deviation (SD) was calculated from the emission peak at 478 nm. Then, according to the formula, the detection limit = 3SD/S, where S is the slope of the linear relationship in the fluorescence titration process, and the detection limit of probe **W** for CN^−^ was calculated to be 68.00 nM. Compared with other CN^−^ probes (Supplementary Table S3), probe **W** had the advantage of a lower detection limit.

### Theoretical Calculations and the Possible Mechanism for Detection of CN^−^

**FIGURE 9:**
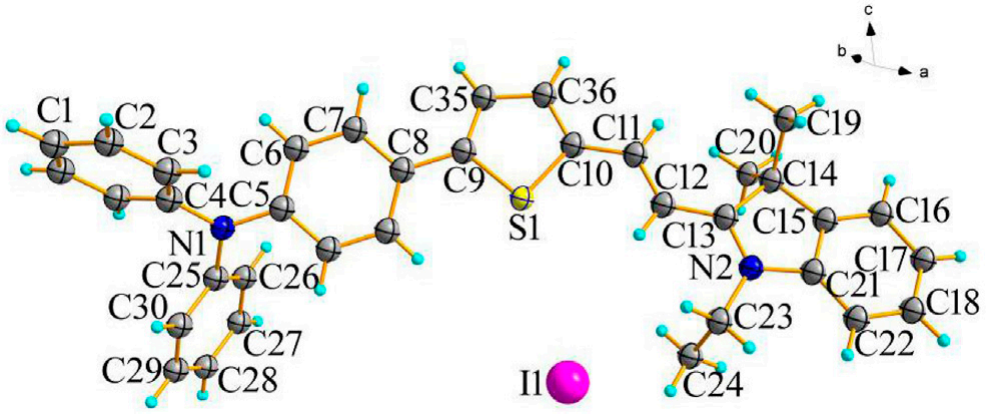
Single crystal X-ray diffraction image of probe W.

Probe **W** (0.1 mmol) was dissolved in 15 ml chloroform, followed by a small amount of 5 ml ethanol. After 5 days of evaporation, appropriately sized x-single crystal diffraction crystals were obtained. Crystal diffraction experiments were performed on the obtained crystals (as shown in Figure 9). The crystal structure shows that 5-(4-(diphenylamine)phenyl)thiophen-2-formaldehyde and 1-ethyl-2,3,3-trimethylindole iodide are bridged by the C=C double bond, which has an E-type configuration. The dihedral angle of the thiophene and pyrrole rings is 167.72°.

**FIGURE 10:**
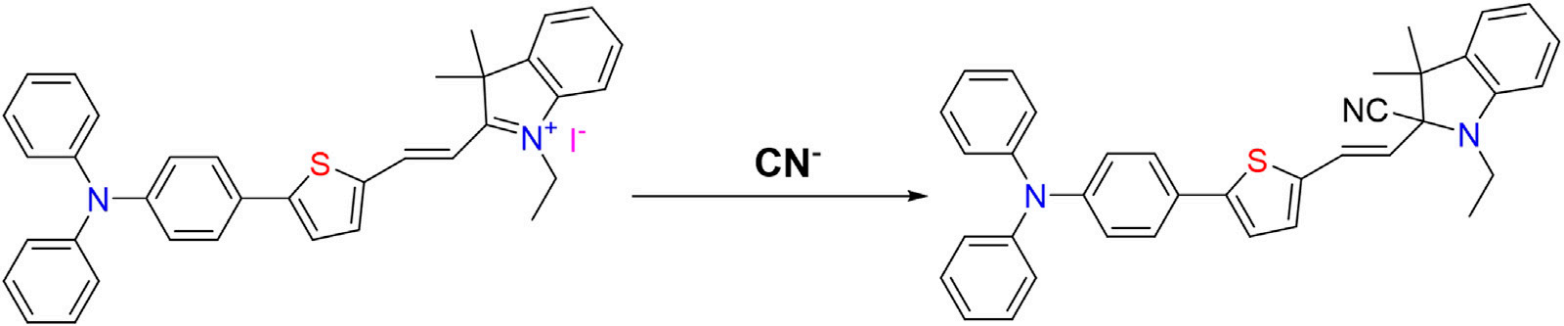
Proposed sensing mechanism of probe W for CN^−^.

According to the mechanism of CN^−^ recognition by fluorescent probes reported in the literature (Ishii et al., 1998; Davis et al., 2014) and combined with the aforementioned experimental results, we inferred the reaction process of CN^−^ recognition by probe **W** is as shown in Figure 10.

**FIGURE 11:**
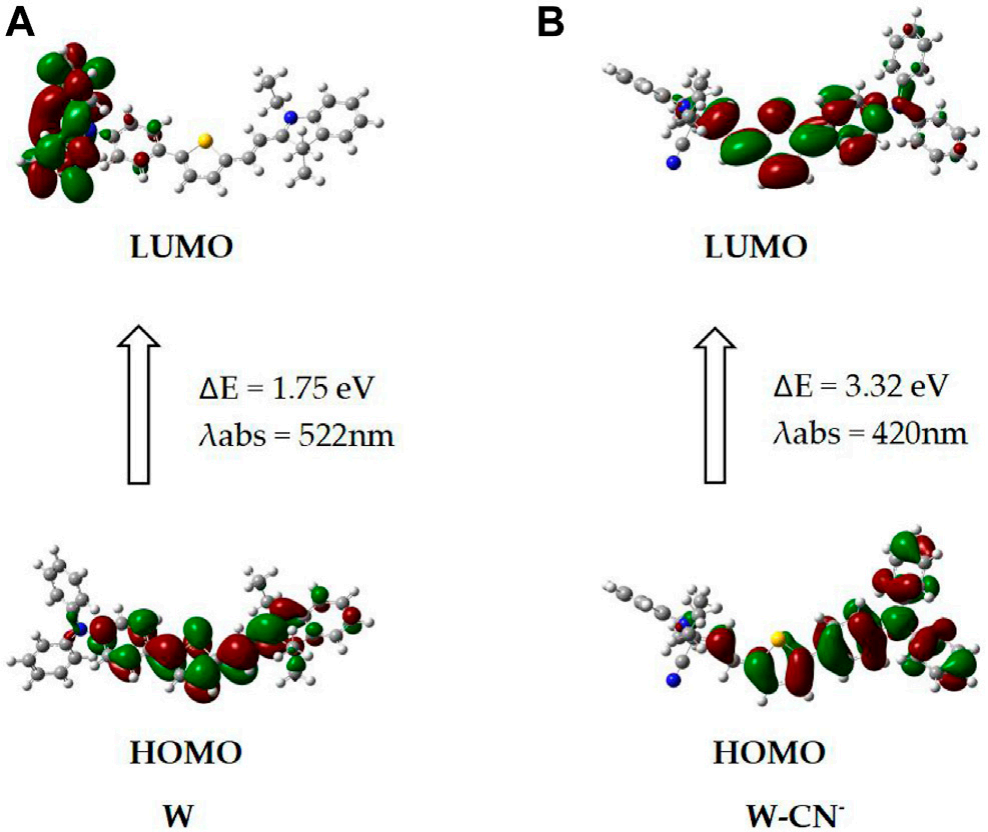
DFT calculation of W and W–CN^−^.

Density functional theory (DFT) quantum chemical calculation on W, its CN^−^ adduct was carried out. Theoretical calculations were performed through B3LYP/6-31G(d) and TDB3LYP/6-31G(d) methods. As depicted in Figure 11, the HOMO-LUMO of W has an energy level of 1.75 eV, and the HOMO-LUMO of W-CN - has an energy level of 3.32 eV. W showed charge transfer character from indole iodide to diphenylamine under excitation, and the calculated absorption peak was 522 nm. However, after the bonding with CN^−^, charge transfer is limited on thiophene moiety. The absorption peak was calculated to be 420 nm and showed a blue shift of 102 nm relative to the absorption of W. Experiments demonstrated that the absorption peak changed from 576 to 382 nm after the probe responded to CN^−^.

**FIGURE 12:**
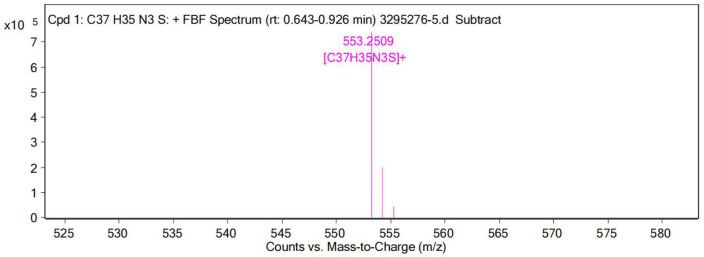
High-resolution mass spectra (HRMS) of the reaction product of probe W upon the addition of CN^−^.

**FIGURE 13:**
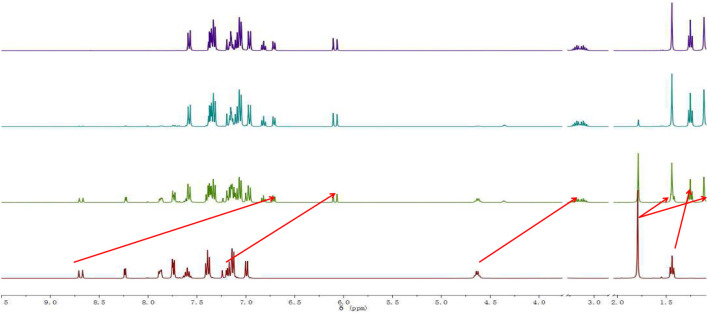
NMR spectroscopic titration of the reaction product of probe W upon the addition of CN^−^.

On gradual addition of CN^−^, the maximum absorption peak at 576 nm of the UV-Vis absorption spectrum of the probe decreased gradually, and the fluorescence intensity at 478 nm increased gradually (Figure 7). The nucleophilic addition reaction between CN^−^ and the positively charged indole salt generates neutral compounds, resulting in a decrease in the electron-absorbing capacity of the indole group, resulting in a decrease in the maximum absorption peak at 576 nm and an increase in the absorption peak at 383 nm. Meanwhile, the conjugated system is destroyed, which inhibits the ICT process and finally presents a fluorescence-enhanced response to CN^−^(Figure 7). The reaction in solution between probe **W** and CN^−^ was verified by a high-resolution mass spectrometry (as shown in Figure 12). [C_37_H_35_N_3_S]^-^ : the theoretical value was 551.2395, and the measured value was 553.2509. In the NMR spectra of the probe TPA-CN, the Hb of the olefin double bond appeared as a double peak at 8.67 ppm. Ha showed a double peak at 7.20 ppm, while the methyl Hc appeared as a single peak at 1.79 ppm. Hd of the ethyl group exhibited a quadruple peak at 4.63 ppm, while He appeared as a triplet peak at 1.44 ppm. When the probe TPA-CN reacted with CN^−^, the Hb and Ha of the olefin double bond shifted to 6.70 and 6.07 ppm, respectively. Methyl Hc was in a different chemical environment and transformed into two single peaks at 1.44 ppm and 1.11 ppm. Hd on the ethyl group was converted to a multi-peak with a shift of 3.15 ppm and He shifted to 1.25 ppm. All the above protons move to higher field after the interaction with CN^−^, which fully indicates that the electron cloud density increases after the interaction, confirming that the reactive imine carbon disrupts the conjugated structure of probe molecules after the addition of CN^−^, and the accompanying deprotonation makes the positively charged indole salt group loose its strong electron-absorbing property. The NMR spectroscopic titration is shown in Figure 13. This work provides a new strategy for the practical application of small-molecule probes in the field of anion detection.

### Applications

To further evaluate the potential application of probe **W** for the detection of CN^−^ in real specimens, water samples from an artificial lake of Guizhou Medical University and running water from our laboratory were collected for detection. The procedure was as follows: 5.90 ml EtOH solution, 100 μL probe reserve solution (20 μM), 2 ml PBS solution, and 2 ml water sample (filtered) were added to a volumetric flask and shaken well. At the same time, the other water sample was treated in the same way, and an appropriate amount of the standard substance (CN^−^) was added. After standing for 80 min, the ratio of the absorption peak at 382 nm to absorption peak at 576 nm and fluorescence intensity at 478 nm of the sample were recorded for further calculations. As shown in Table 1, the recovery rates of the probe were 100.17—100.0.86% in artificial lake water and 100.54–101.64% in running water by UV-Vis absorption spectroscopy. As shown in Table 2, the recovery rate of the probe in artificial lake water was 99.42–100.71% and 100.59–101.17% in running water by fluorescence spectroscopy. The results show that **W** is a sensitive and highly selective probe for monitoring environmental water samples.

**TABLE 1:** Detailed data for CN^−^ detection in real water samples.

Sample	Measured	Added	Detected	Recovery	RSD
	(μmol·L^−1^)	(μmol·L^−1^)	(μmol·L^−1^)	(*n* = 3, %)	(*n* = 3, %)
Artificial lake	0.48	10.00	10.57	100.86	0.10
		20.00	20.63	100.73	0.30
		35.00	35.54	100.17	0.20
Running water	0.34	10.00	10.51	101.64	0.70
		20.00	20.49	100.74	0.10
		35.00	35.53	100.54	0.50

**TABLE 2:** Detailed data for CN^−^ detection in real water samples.

Sample	Measured	Added	Detected	Recovery	RSD
	(μmol·L^−1^)	(μmol·L^−1^)	(μmol·L^−1^)	(*n* = 3, %)	(*n* = 3, %)
Artificial lake	0.39	10.00	10.33	99.42	0.10
		20.00	20.51	100.59	0.20
		35.00	35.64	100.71	0.40
Running water	0.22	10.00	10.34	101.17	0.10
		20.00	20.34	100.59	0.20
		35.00	35.60	101.08	0.10

## Conclusion

In summary, a novel fluorescent probe based on 5-(4-(diphenylamine) thiophen-2-formaldehyde was developed by a two-step method. In addition, in the presence of CN^−^ ions, the probe solution changed from blue to nearly colorless under sunlight, and from dark to bright under a UV lamp. The detection limit was as low as 0.48 μM by UV-Vis absorption spectroscopy, and 68.00 nM by fluorescence spectroscopy. This indicates that probe **W** may be used for visual and instrumental detection of CN^−^. Titration experiments show that the probe has good linearity and can be used for quantitative and qualitative determination of CN^−^ in real samples. We believe that not only does this work provide a new example of a small molecule probe for ion detection but that these results may inform researchers in the broader field of cell imaging, which is ongoing in our lab.

## Data Availability

The datasets presented in this study can be found in online repositories. The names of the repository/repositories and accession number(s) can be found below: https://www.ccdc.cam.ac.uk/, CCDC:2143454.
