# Supplementary material for: A Turn-On Fluorescent Chemosensor for Cyanide Ion Detection in Real Water Samples
Source: Front Chem. 2022 Jul 18;10:923149. doi: 10.3389/fchem.2022.923149 (PMC9339681; doi:10.3389/fchem.2022.923149)
Supplement: Supplementary file 1 [file DataSheet2.pdf]

Supplementary Material

A turn-on fluorescent chemosensor selectively detects cyanide in real water samples

Qing Shi [1,2], Shou-Ting Wu[1], Lingyi Shen[1], Tao Zhou[1], Hong Xu[1], Zhi-Yong Wang[1], Xian-Jiong Yang[1,*], Ya-Li Huang[1], Qi-Long Zhang[1,*]

[1] School of Public Health, the key Laboratory of Environmental Pollution Monitoring and Disease Control, Ministry of Education, Guizhou Medical University, Guiyang 550004, China.

[2] The Second Affiliated Hospital of Guizhou University of Traditional Chinese medicine, China.

* **Correspondence:** Qi-Long Zhang

sciqlzhang@gmc.edu.cn

Fax: +86-139-8415-8601 (Q.L.Z.)

Xian-Jiong Yang

yangxianjiong@126.com;

Fax: +86-135-9517-4501 (X.J.Y.)

1 Adsorption Figures

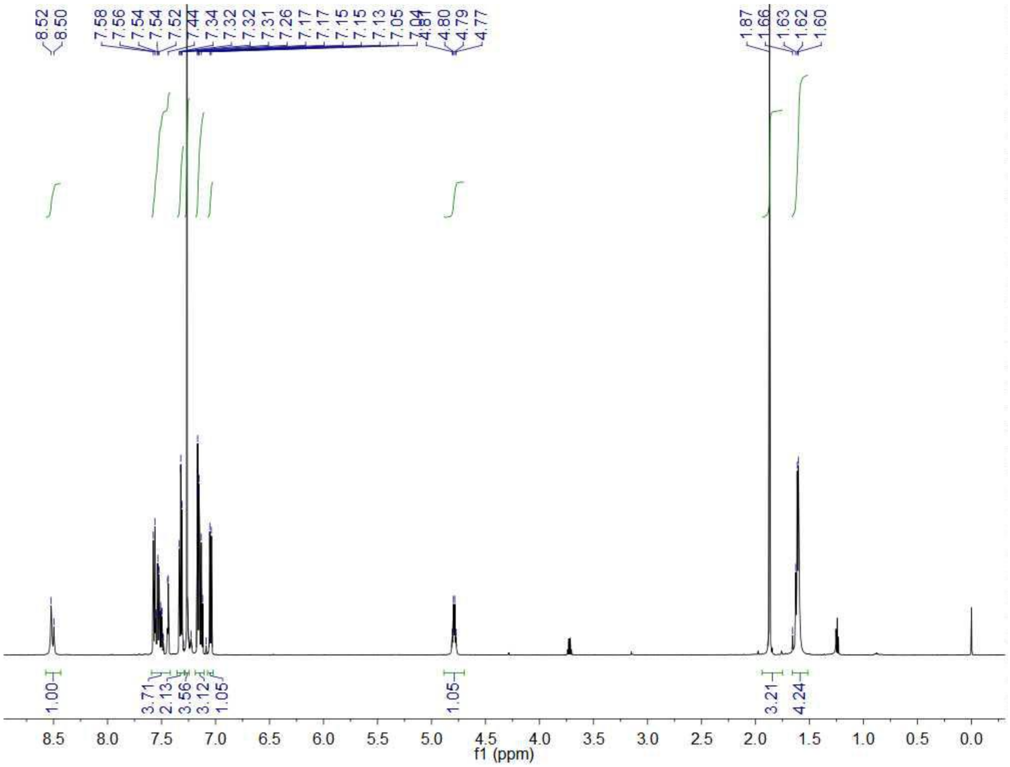

Supplementary Figure 1. ^{1}H NMR spectrum of probe **W.**

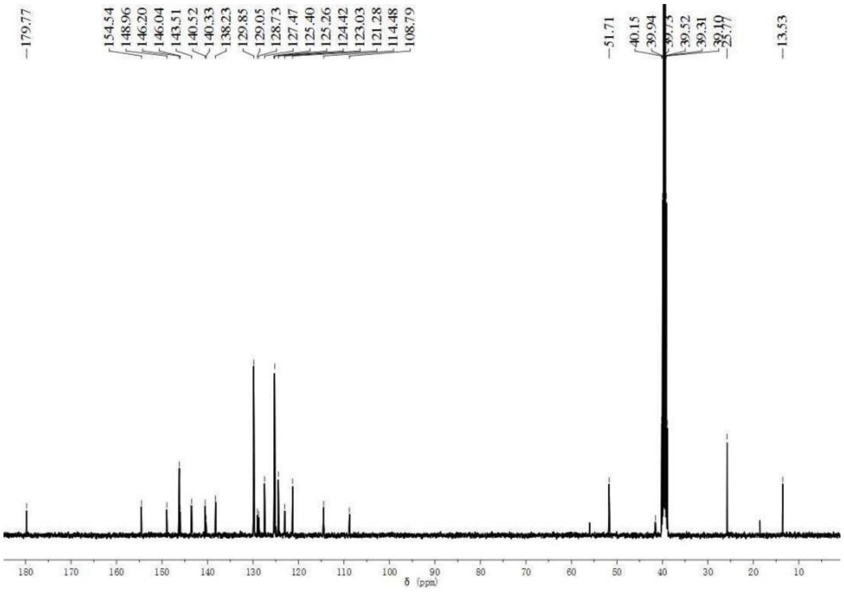

Supplementary Figure 2. ^{13}C NMR spectrum of the reaction product of probe **W** upon the addition of CN^- .

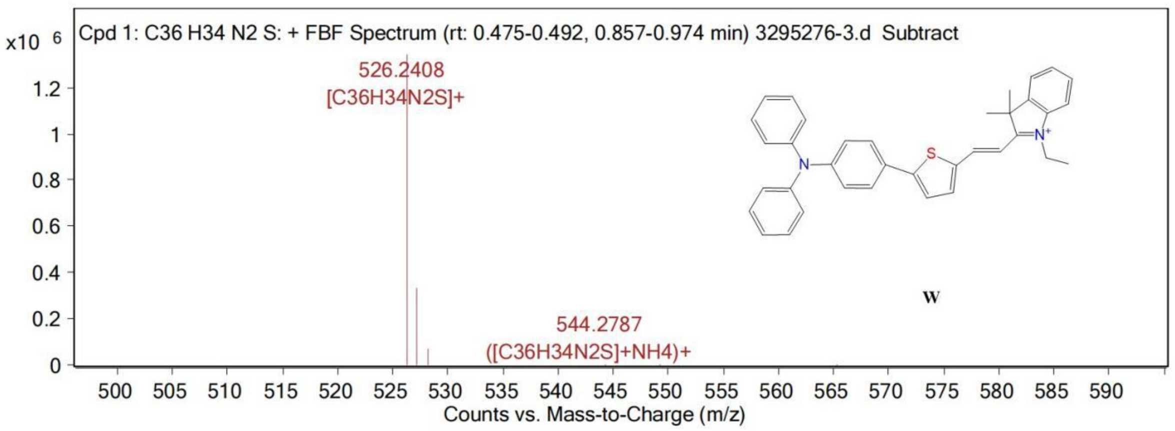

Supplementary Figure 3. HRMS spectrum of probe W.

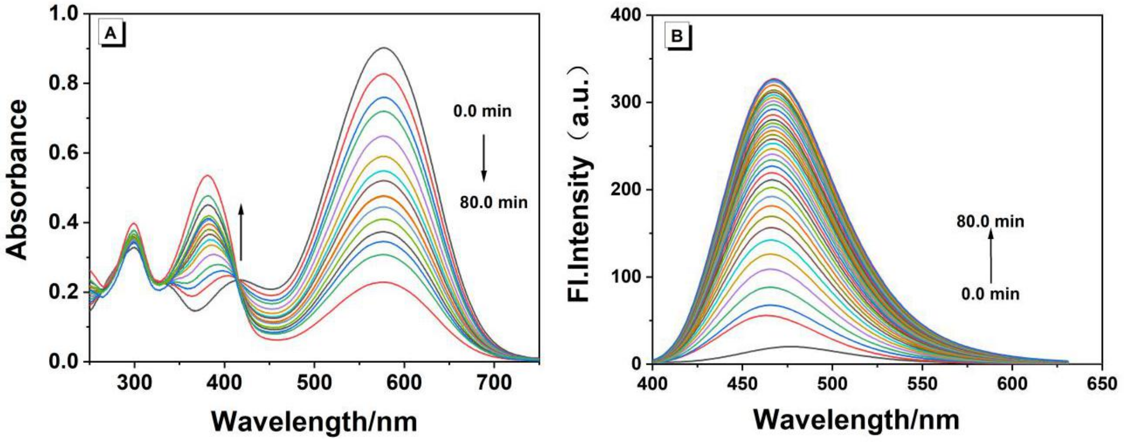

Supplementary Figure 4. (A) Uv-vis absorption spectra; (B) and fluorescence spectra of probe W (20 μM) in EtOH/water (V_{EtOH}/V_{water}=3/2, pH=7.40) after adding CN^- (40 μM) over time (λ ex/λ em = 378/478 nm, slit: 5/5 nm, voltage: 500 v).

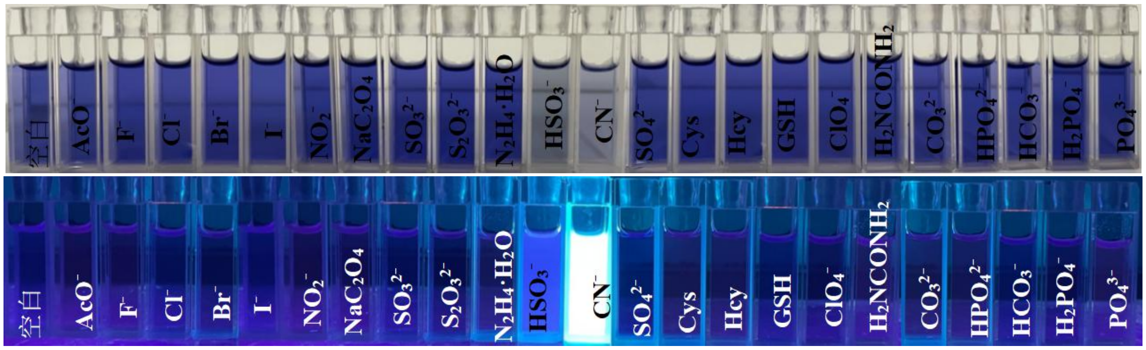

Supplementary Figure 5. Photographs of probe **W**-anion complex in EtOH/water (V_{EtOH}/V_{H2O}=3/2, pH=7.40) solution under (A) natural light and (B) 365 nm UV lamp.

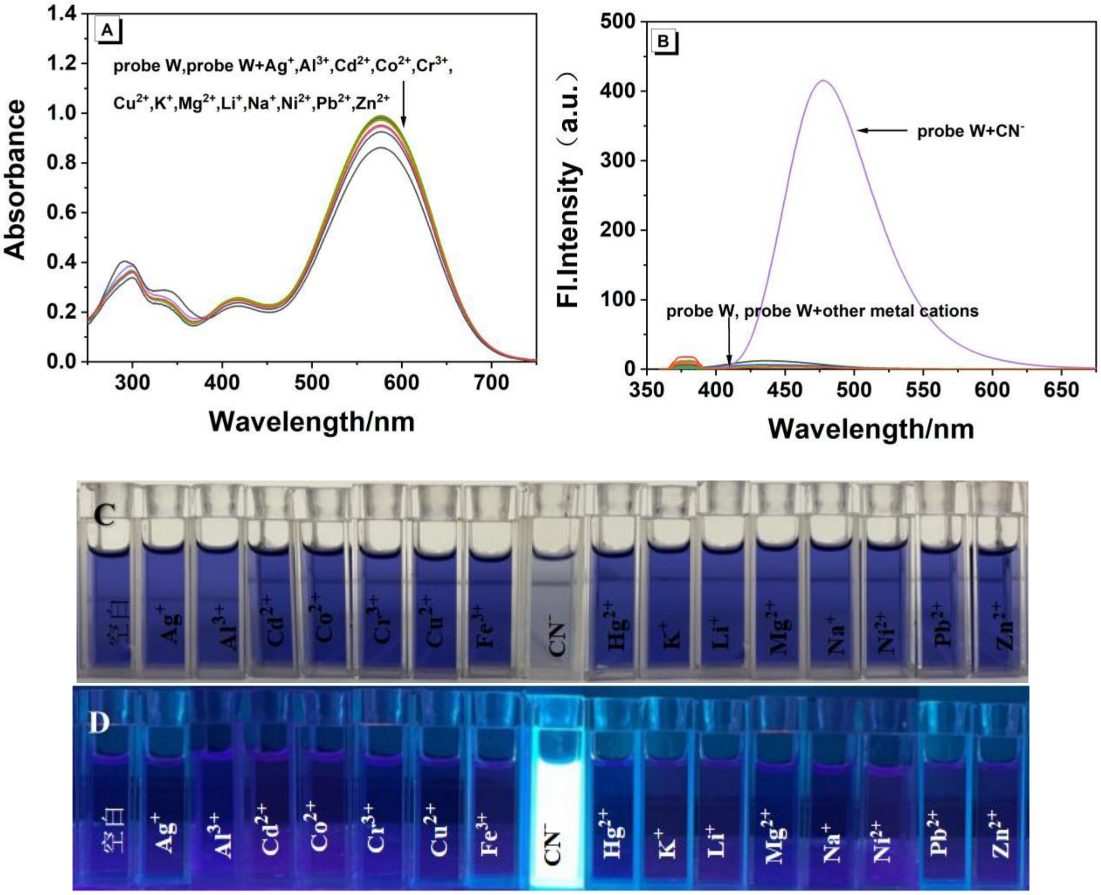

Supplementary Figure 6. (A) UV-vis and (B) Fluorescence spectra of the fluorescence probe **W** interacting with different cations ($\lambda_{ex}/\lambda_{em}$ = 378/478 nm, slit: 5/5 nm, voltage: 500 V). Photographs of probe **W**-cation complex in EtOH/water (V_{EtOH}/V_{H2O} = 3/2, pH=7.40) solution under (A) natural light and (B) 365 nm UV lamp.

2 Supplementary Tables

Supplementary Table 1. Summary of crystal data of probe **W**

Parameter	Probe
Empirical formula	$C_{36}H_{33}IN_2S$
Formula weight [g mol^{-1}]	652.60
Crystal system	monoclinic
Space group	*P2₁/c*
a [Å]	20.223(2)
b [Å]	10.0554(11)
c [Å]	17.0768(19)
β [°]	114.740(3)
Volume [Å^3]	3153.8(6)
Z	4
Density, calcd [gm^{-3}]	1.374
Temperature [K]	273(2)
F(000)	1328
Unique reflns	3107
Obsdreflns	*7519*
Parameters	364
R_{int}	0.0726
$R[I>2\sigma(I)]^a$	0.0565

W[all data]R^b	0.1387
GOF on F^2	0.999

[a] Conventional R on F_{hkl}: $\Sigma||F_o|-|F_c||/\sigma|F_o|$. [b] Weighted R on $|F_{hkl}|^2$: $\Sigma[w(F_o^2-F_c^2)^2]/\Sigma[w(F_o^2)^2]^{1/2}$.

Supplementary Table 2. The ratio of absorption peak at 382 nm to absorption peak at 576 nm of probe **W** (20 μM) and **W**-CN^- (40 μM) complex

versus different pH value within 1440 mins.

	**pH value**	**Time**			
		**0 min.**	**360 min.**	**720 min.**	**1440 min.**
	**4**	**0.1650**	**0.1652**	**0.1651**	**0.1653**
	**5**	**0.1665**	**0.1668**	**0.1667**	**0.1669**
	**6**	**0.1663**	**0.1666**	**0.1664**	**0.1667**
	**7**	**0.1656**	**0.1659**	**0.1658**	**0.1661**
**Probe W**	**8**	**0.1689**	**0.1690**	**0.1691**	**0.1692**
	**9**	**0.1693**	**0.1696**	**0.1698**	**0.1699**
	**10**	**0.1737**	**0.1739**	**0.1741**	**0.1742**
	**11**	**0.1743**	**0.1744**	**0.1746**	**0.1747**
	**4**	**0.9348**	**0.9351**	**0.9352**	**0.9355**
	**5**	**1.1043**	**1.1046**	**1.1047**	**1.1049**
	**6**	**1.2495**	**1.2496**	**1.2497**	**1.2499**

	**7**	**1.3740**	**1.3744**	**1.3745**	**1.3747**
**W-CN$^-$ complex**	**8**	**1.5794**	**1.5795**	**1.5796**	**1.5799**
	**9**	**1.6925**	**1.6927**	**1.6929**	**1.6931**
	**10**	**1.4150**	**1.4153**	**1.4155**	**1.4156**
	**11**	**1.6904**	**1.6905**	**1.6907**	**1.6908**

Supplementary Table 3. The fluorescence intensity (a.u.) of probe **W** (20 μM) and **W**-CN$^-$ (40 μM) complex

versus different pH value within 1440 mins (478 nm, slit: 5/5 nm, voltage: 500 v).

	**pH value**	**Time**			
		**0 min.**	**360 min.**	**720 min.**	**1440 min.**
	**4**	**0.2090**	**0.2092**	**0.2094**	**0.2096**
	**5**	**0.4649**	**0.4652**	**0.4653**	**0.4653**
	**6**	**0.1176**	**0.1176**	**0.1177**	**0.1178**
	**7**	**0.2750**	**0.2753**	**0.2754**	**0.2756**
**Probe W**	**8**	**0.6562**	**0.6564**	**0.6564**	**0.6565**
	**9**	**0.9400**	**0.9403**	**0.9404**	**0.9406**
	**10**	**1.6307**	**1.6308**	**1.6311**	**1.6312**
	**11**	**3.0471**	**3.0473**	**3.0475**	**3.0479**
	**4**	**275.3837**	**275.3839**	**275.3841**	**275.3842**

	**5**	**308.6688**	**308.6689**	**308.6691**	**308.6693**
	**6**	**317.0682**	**317.0687**	**317.0689**	**317.0692**
	**7**	**329.7695**	**329.7697**	**329.7699**	**329.7703**
**W-CN$^-$ complex**	**8**	**333.5925**	**333.5926**	**333.5929**	**333.5932**
	**9**	**343.8035**	**343.8036**	**343.8038**	**343.8038**
	**10**	**341.7222**	**341.7227**	**341.7229**	**341.7235**
	**11**	**364.4769**	**364.4772**	**364.4773**	**364.4775**

Supplementary Table 4. Comparison data with reported CN$^-$ sensors

Structure	Fluorescence	solvent	Detection limit
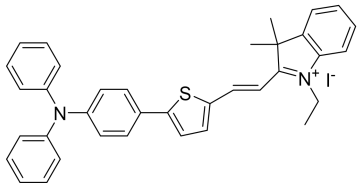 **Our work**	Turn-on	EtOH/ H_2O (3:2)	0.48 μM/ 68.00 nM
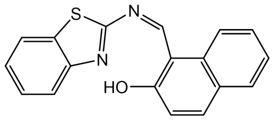 **Probe 1** (You et al., 2014)	Turn-off	DMSO/ H_2O (8:2)	0.78 μM

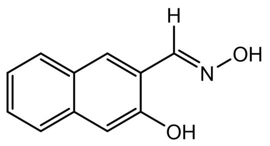 **Probe 1** (Wang et al., 2015)	Turn-on	MeOH/ H_2O (1:9)	0.18 µM
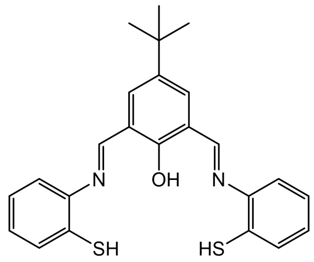 **Probe R** (Kumar et al., 2019)	Turn-on	DMSO/ H_2O (2:8)	0.96 µM
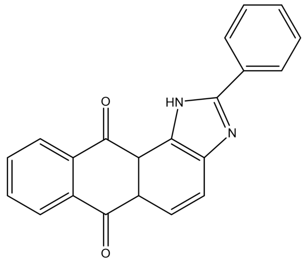 **Probe A** (Sukdeb et al., 2010)	Turn-on	acetonitrile/ HEPES (1:1)	1.42 µM
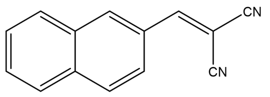 **Probe 1** (Long et al., 2019)	Turn-on	DMF/H_2O (6:4)	0.23 µM
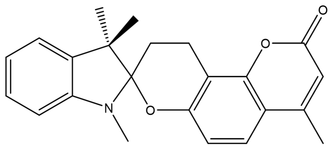	Turn-on	MeCN/ H_2O (3:7)	1.00 µM

**Probe 2** (Shiraishi et al., 2016)			
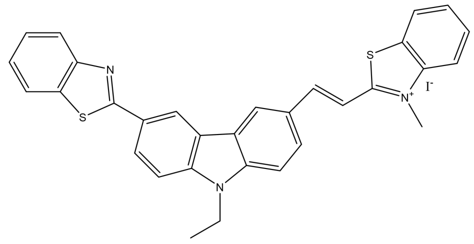 **BCB** (Sun et al., 2016)	Turn-on	DMSO/H_2O (1:9)	0.09 μM
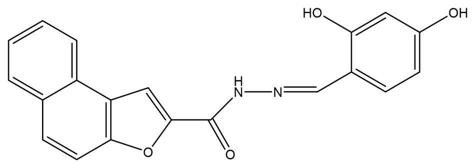 **Q1-3** (Qu et al., 2016)	Turn-on	DMSO/ H_2O (1:9)	0.81 μM

3 References

You, G. R., Park, G. J., Lee, S. A., Choi, Y. W., Kim, Y. S., Lee, J. J., Kim, C. (2014). A single chemosensor for multiple target anions: The simultaneous detection of CN^- and OAc^- in aqueous media. *Sens Actuators B*, *202*, 645-655. doi: http://dx.doi.org/10.1016/j.snb.2014.05.124.

Wang, S. T., Chir, J. L., Jhong, Y., Wu, A. T. (2015). A turn-on fluorescent sensor for detection of cyanide in aqueous media. *J. Lumin.*, *167*, 413-417. doi: http://dc.doi.org/10.1016/j.jlumin.2015.06.046.

Kumar, P. S., Lakshmi, P. R., Elango, K. P. (2019). An easy to make chemoreceptor for the selective ratiometric fluorescent detection of cyanide in aqueous solution and in food materials. *New J. Chem.*, *43* (2), 675-680. doi: 10.1039/C8NJ05587D.

Sukdeb, S., Amrita, G., Prasenjit, M., Sandhya, M., Sanjiv, K. (2010). Mishra, E. Suresh.; Satyabrata,

D.; Amitava, D., Specific Recognition and Sensing of CN^{-} in Sodium Cyanide Solution. *Org. Lett.*, *12*, 15, 3406-3409. doi: 10.1021/ol101281x.

Long, L. L., Yuan, X. Q., Cao, S. Y., Han, Y. Y., Liu, W. G., Chen, Q., Han, Z. X., Wang, K. (2019). Determination of Cyanide in Water and Food Samples Using an Efficient Naphthalene-Based Ratiometric Fluorescent Probe. *ACS Omega*, *4,* 6, 10784-10790. doi: 10.1021/acsomega.9b01308.

Shiraishi, Y., Nakamura, M., Hayashi, N., Hirai, T. (2016). Coumarin–Spiropyran Dyad with a Hydrogenated Pyran Moiety for Rapid, Selective, and Sensitive Fluorometric Detection of Cyanide Anion. *Anal. Chem.*, *88*, 13, 6805-6811. doi: 10.1021/acs.analchem.6b01279.

Sun, X. Y., Wu, Y., Deng, X. H., Zhang, J. F., Zhao, Z. (2016). A colorimetric and ratiometric fluorescent probe for the selective detection of cyanide anions in aqueous media and living cells. *RSC Adv.*, *6* (13), 10266-10271. doi: 10.1039/C5RA26587H.

Qu, W. J., Li, W. T., Zhang, H. L., Wei, T. B., Lin, Q., Yao, H., Zhang, Y. M. (2016). Rapid and Selective Detection of Cyanide Anion by Enhanced Fluorescent Emission and Colorimetric Color Changes at Micromole Levels in Aqueous Medium. *J. Heterocycl. Chem.* **2016**, 115-124. doi: https://doi.org/10.1002/jhet.3113.
